# Incidence of cutaneous malignant melanoma by socioeconomic status in Canada: 1992–2006

**DOI:** 10.1186/s40463-015-0107-1

**Published:** 2015-12-02

**Authors:** Stephanie E. Johnson-Obaseki, Varant Labajian, Martin J. Corsten, James T. McDonald

**Affiliations:** Department of Otolaryngology-Head and Neck Surgery, University of Ottawa, S3 – 501 Smyth Road, Ottawa, ON K1H 8L6 Canada; Department of Otolaryngology – Head and Neck Surgery, Aurora Health Care, Aurora St. Luke’s Medical Center, 2801 W. Kinnickinnic River Parkway, Suite 630, Milwaukee, WI 53215 USA; Department of Economics, University of New Brunswick, PO Box 4400, Fredericton, NB E3B6C4 Canada

**Keywords:** Melanoma, Socioeconomic status, Income, Incidence, Urban residence, Rural residence

## Abstract

**Background:**

There are no nationwide studies documenting changes in cutaneous malignant melanoma incidence or association of incidence with socioeconomic status (SES) in Canada. We sought to determine whether melanoma incidence increased from 1992 to 2006 and if there was an association between SES and melanoma incidence. Additionally, we studied whether there was a correlation between province of residence and melanoma incidence.

**Methods:**

Cases from the Canadian Cancer Registry were reviewed. Demographic and socioeconomic information were extracted from the Canadian Census of Population data. Cases were linked to income quintiles by postal code. A negative binomial regression was performed to identify relationships among these variables.

**Results:**

Overall incidence of melanoma in Canada increased by 67 % from 1992 to 2006 (*p* < 0.0001). The increase in incidence was greater for melanoma in situ compared with invasive melanoma (136 % versus 52 % [*p* < 0.0001]). Incidence was positively correlated with higher income quintiles; the incidence rates among patients in the lowest income quintiles were 67 % of that for the highest income quintiles (*p* < 0.0001).

**Discussion:**

A wide variety of explanations have been postulated for an increased incidence in melanoma among persons of higher SES, including access to and awareness of screening, more access to vacations in sunny climates, and increased leisure time. Variations in incidence of melanoma by urban vs. rural location and province may indicate differences in access to dermatologists across Canada.

**Conclusions:**

Melanoma incidence is increasing in Canada and is higher among people in high SES groups. This rise is likely due to a combination of factors including a true rise in incidence due to increases in sun exposure, and also an increased detection rate, particularly in those who are more aware of the disease and have access to resources for detection.

## Background

Worldwide incidence of cutaneous malignant melanoma (hereafter referred to as ‘melanoma’) has continued to rise over the past several decades, with about 160,000 new cases diagnosed each year in the United States alone [1–3]. Most of the increased incidence has occurred in countries with predominately fair-skinned people. The countries with the highest incidence include Australia [4], New Zealand [5], Western European countries [6] and North American countries [7, 8]. Though melanoma is less common than other skin cancers, it causes 75 % of deaths from skin cancer [2].

Ongoing depletion of the ozone layer and increased solar ultraviolet radiation reaching the earth’s surface is causing a continued rise in skin cancer incidence. The World Health Organization estimates that there is an increase of 4500 skin cancer cases for each 10 % decrease in ozone levels [1]. The risk of melanoma is associated with increases in sun holidays [9, 10] and use of indoor tanning beds [11, 12]. Earlier detection of thinner melanomas accounts for a substantial proportion of the increase in melanoma incidence [13–15]. This can likely be explained by improved education and screening strategies [16]. As melanoma results from cumulative exposure to the sun, people have more time to accumulate sun damage and subsequent cancers with increased longevity [17, 18].

Socioeconomic status (SES) is known to be associated with both melanoma incidence and survival [10, 19–21]. High SES is associated with increased incidence of melanoma, thinner tumors, increased survival and decreased mortality [19–21]. Singh et al. examined the incidence of cutaneous melanoma in the United States by merging population-based central cancer registries with county-level SES estimates from the U.S. Census Bureau [21]. They found that counties with lower poverty, higher education, higher income and lower unemployment had higher age-adjusted melanoma incidence rates for early stage disease [21]. Similarly, Perez-Gomez et al. examined the association of SES and melanoma in Sweden using the Swedish Cancer Environment Registry and the Background Population Registry, which contain information on occupation, residence and date of death [20]. They used these data to determine association between SES (using occupation as a proxy) and melanoma and found a marked increase in the risk of melanoma in white-collar workers, particularly for men [20]. Other studies have yielded similar results. Theories explaining the increased incidence of melanoma among higher SES individuals include intense intermittent ultraviolet exposure (sun holidays) [20] and increased knowledge of and access to screening [16]. The reason for decreased mortality in this group may be related to an earlier stage at diagnosis [16].

Haider et al. used a population-based, cross-sectional study of administrative health care databases in a single Canadian province (Ontario) to determine if there was an association between melanoma prevalence and income level (used as a proxy for SES) [22]. They found an increased prevalence of 225 % in the highest compared with the lowest income groups. The Western Canada Melanoma study was performed by Gallagher et al. to determine the association between SES and risk of melanoma in four Canadian provinces (British Columbia, Alberta, Saskatchewan and Manitoba) [23]. Their study consisted of a detailed, multivariate analysis of 261 male cases as well as age- and sex-matched controls, using usual occupation as their proxy for SES. In their univariate analysis they found a strong positive association between SES and risk of melanoma. However, using multivariate analysis, they found that this association was substantially explained by host constitutional factors and sunlight exposure.

There has been conflicting evidence in the literature regarding how urban or rural residence affects the incidence of melanoma. Aase et al. used Norwegian Cancer Registry data from 1955 to 1989 and found urban residence to be associated with high melanoma incidence [24]. Conversely, Wesseling et al. analyzed the Costa Rica Cancer Registry data from 1981 to 1993 and found an increase in melanoma incidence in rural areas [25]. Perez-Gomez et al. also included an analysis of melanoma incidence by urban versus rural place of residence [20]. They found that there was an increased risk in men living in larger towns. The pattern for women living in urban areas showed an increase only in melanomas of the leg. Increase in intermittent sun exposure (a known significant risk factor for melanoma) and, perhaps, improved access to health care for screening and detection may cause increased incidence [20].

To our knowledge, there has not been a Canadian study using nationwide Cancer Registry data to examine the relationship between melanoma cancer incidence, SES and geographic location in Canada. The purpose of this study was to determine whether there was an increased incidence of melanoma in Canada from 1992 to 2006 and if there was an association between SES and incidence. Additionally, we studied whether there was an association between urban versus rural residence and incidence of melanoma and if there was a correlation between province of residence and incidence of cutaneous melanoma.

## Methods

Data for this study were extracted from the Canadian Cancer Registry data file and the Canadian Census of Population from Statistics Canada. The registry data file contains patient demographic and tumor-specific information on each tumor included in provincial and territorial cancer registries from 1992 to 2006 inclusively, while the census files provide neighborhood-level (dissemination area, DA) data on age/sex composition, average household income and location of residence. The reason that newer data is not included is due to the fact that 2006 is the last year that the long form census was used. Data sources, the methods employed to generate income quintiles and demographic characteristics, and the methods used to construct the dataset for estimation have been described in detail elsewhere [26, 27]. Since census data are available only every 5 years, cases diagnosed in 1992–1995 were associated with data from the 1991 census year (CY); cases diagnosed in the 1996–2000 period, the 1996 CY; cases diagnosed in the 2001–2005 period, the 2001 CY; and cases diagnosed in the 2006–2007 period, the 2006 CY. The number of DAs for which census information was available was 32,825 in 1991, 38,016 in 1996, 46,909 in 2001 and 52,443 in 2006. Income quintile (InQ) for each DA was defined relative to other DAs in the associated census division, which Statistics Canada defines as a group of neighboring municipalities joined together for the purposes of regional planning and managing of common services. Dissemination areas within each census division were sorted by average household income then assigned to one of five InQs. All analysis was within the University of New Brunswick Research Data Centre (NB-RDC) and all output was vetted for release using enhanced vetting methods required by Statistics Canada. Ethics approval is not required for research projects using data stored in the NB-RDC.

For each CY, the unit of observation for the analysis of incidence was the DA, and the key variable of interest was the number of cases of melanoma (International Classification of Diseases codes: O2/3 C44.0-C44.9), including both malignant melanoma and melanoma in situ, diagnosed in adults over the age of 18 in each DA over a relevant period of time corresponding to the CY. Unfortunately, data on in situ melanoma cases are not available for Ontario in the data provided by Statistics Canada, which necessitates estimation of the main model for a number of alternative sample specifications. These include: 1) all cases of melanoma, invasive melanoma and in situ melanoma for all provinces and territories except Ontario and 2) invasive melanoma for all provinces and territories including Ontario.

The exposure variable was the adult population in the DA during the CY multiplied by the number of years in the corresponding time interval for that census (2, 4 or 5 years). Negative binomial regression models were estimated where neighborhood SES, as measured by the InQ of the DA, was captured by a 0/1 binary variable for each InQ, with the highest InQ specified as the baseline. Indicator variables for each CY from 1996 to 2006 with 1991 as the reference year were included to reflect changes over time. The regressions also included indicator variables for province of residence of the individual at the time of diagnosis as well as whether the DA of residence was a larger urban center (census metropolitan area with a total population of at least 100,000 of which 50,000 or more must live in an urban core), smaller urban center (census agglomeration with a total population of between 10,000 and 100,000) and rural, if the DA was not located in either a census metropolitan area or census agglomeration.

To control for differences in the ethnic/racial composition across DA populations and over time, the total adult population in each DA that identify as 1) black, 2) south Asian, 3) east Asian and 4) other visible minority group were extracted from each census file. Each of these measures was included as controls in the analysis.

## Results

### Incidence by census year

Using multivariate regression, the incidence of invasive melanoma increased with time over the study period of CY 1991 to CY 2006 after controlling for age, sex and race (Table 1). For invasive melanoma (excluding Ontario) the incidence rate ratio (IRR) increased from 1.0 in the reference CY (1991) to 1.52 in the 2006 CY (IRR 1.52, *p* < 0.000, 95 % confidence interval [CI] 1.46–1.59). Most of the increase in incidence occurred between the 2001 and 2006 CYs (IRR 2001 1.05, *p* < 0.00), 95 % CI 1.01–1.09). The results were not materially different when Ontario was included in the analysis. For melanoma in situ, a similar but more pronounced pattern of increasing IRRs with later CYs was identified, with the 2006 CY showing an incidence rate 136 % greater than the 1991 CY (IRR 2.36, *p* < 0.000, 95 % CI 2.17–2.57). Table 2 displays the multivariate analysis regression results broken down by controls for the age/sex composition of the DA as well as controls for the various race proportions in the DA.

**Table 1:** Incidence rate ratios of the diagnosis of melanoma^a^ (excludes Ontario)^b^

	All melanoma (*n* = 105,681)			In situ			Invasive		
	IRR	*P* value	95 % CI	IRR	*P* value	95 % CI	IRR	*P* value	95 % CI
Place of residence									
City (census metropolitan area)	1			1			1		
Town (census agglomeration)	0.80	0.00	(0.77–0.82)	0.69	0.00	(0.65–0.74)	0.82	0.00	(0.79–0.85)
Rural	0.80	0.00	(0.78–0.82)	0.71	0.00	(0.67–0.75)	0.82	0.00	(0.79–0.85)
Income quintile									
Highest	1			1			1		
2^nd^ highest	0.89	0.00	(0.86–0.91)	0.86	0.00	(0.81–0.92)	0.89	0.00	(0.86–0.92)
Middle	0.85	0.00	(0.83–0.88)	0.79	0.00	(0.74–0.85)	0.86	0.00	(0.83–0.90)
2^nd^ lowest	0.83	0.00	(0.81–0.86)	0.76	0.00	(0.71–0.82)	0.85	0.00	(0.82–0.88)
Lowest	0.79	0.00	(0.76–0.81)	0.68	0.00	(0.62–0.73)	0.82	0.00	(0.78–0.85)
Census year									
2006	1.67	0.00	(1.62–1.73)	2.36	0.00	(2.17–2.57)	1.52	0.00	(1.46–1.59)
2001	1.10	0.00	(1.07–1.13)	1.38	0.00	(1.28–1.49)	1.05	0.02	(1.01–1.09)
1996	1.01	0.61	(0.98–1.04)	1.09	0.02	(1.01–1.18)	1.00	0.89	(0.96–1.03)
1991	1			1			1		
Province									
Newfoundland and Labrador	0.67	0.00	(0.63–0.72)	0.73	0.00	(0.63–0.84)	0.66	0.00	(0.61–0.71)
Prince Edward Island	1.16	0.00	(1.06–1.26)	1.59	0.00	(1.31–1.92)	1.07	0.26	(0.95–1.20)
Nova Scotia	1.12	0.00	(1.08–1.17)	1.65	0.00	(1.52–1.79)	1.00	0.94	(0.95–1.05)
New Brunswick	0.99	0.69	(0.95–1.04)	1.20	0.00	(1.08–1.32)	0.94	0.04	(0.89–1.00)
Quebec	0.39	0.00	(0.37–0.40)	0.17	0.00	(0.15–0.18)	0.44	0.00	(0.42–0.45)
Ontario	^b^			^b^			^b^		
Manitoba	0.78	0.00	(0.75–0.81)	0.90	0.03	(0.83–0.99)	0.74	0.00	(0.70–0.78)
Saskatchewan	0.74	0.00	(0.71–0.78)	0.69	0.00	(0.61–0.77)	0.75	0.00	(0.71–0.80)
Alberta	0.95	0.00	(0.92–0.98)	1.36	0.00	(1.27–1.45)	0.85	0.00	(0.82–0.89)
British Columbia	1			1			1		
Territories	0.45	0.00	(0.37–0.57)	0.55	0.02	(0.34–0.90)	0.43	0.00	(0.33–0.56)

*CI* confidence interval, *IRR* incidence rate ratio

^a^Negative binomial regressions on the incidence of diagnosed cases of melanoma by dissemination area (DA) and census year. Regressions include detailed controls for the age/sex composition of the DA as well as controls for the proportions of adults in the DA who are 1) black, 2) South Asian, 3) other Asian and 4) other visible minority groups

^b^Cases of in situ melanoma are not available for Ontario in the Canadian Cancer Registry dataset in the Statistics Canada Research Data Centre so Ontario DAs are excluded from these regressions

**Table 2:** Incidence rate ratios of the diagnosis of melanoma^a^ (excludes Ontario)^b^–other controls

	All melanoma (*n*–105,681)			In situ			Invasive		
	IRR	*P* value	95 % CI	IRR	*P* value	95 % CI	IRR	*P* value	95 % CI
Proportion of Population by age/sex									
Male 20–29	1			1			1		
Male 30–39	0.99	0.01	(0.99–1.00)	0.98	0.01	(0.97–0.99)	1.00	0.25	(0.99–1.00)
Male 40–49	0.99	0.00	(0.98–0.99)	0.98	0.01	(0.97–1.00)	0.99	0.01	(0.98–1.00)
Male 50–59	0.99	0.01	(0.99–1.00)	0.99	0.48	(0.98–1.01)	0.99	0.02	(0.98–1.00)
Male 60–69	1.00	0.42	(0.99–1.01)	1.00	0.99	(0.98–1.02)	0.99	0.34	(0.98–1.01)
Male 70–79	1.01	0.00	(1.01–1.02)	1.01	0.32	(0.99–1.03)	1.01	0.00	(1.01–1.02)
Male 80+	1.02	0.00	(1.02–1.03)	1.04	0.00	(1.02–1.06)	1.02	0.00	(1.01–1.03)
Female 20–29	0.99	0.07	(0.99–1.00)	0.99	0.04	(0.97–1.00)	1.00	0.23	(0.99–1.00)
Female 30–39	1.00	0.56	(0.99–1.00)	1.00	0.91	(0.99–1.01)	1.00	0.39	(0.99–1.00)
Female 40–49	1.01	0.00	(1.01–1.02)	1.02	0.01	(1.00–1.03)	1.01	0.00	(1.01–1.02)
Female 50–59	1.02	0.00	(1.01–1.03)	1.03	0.00	(1.01–1.04)	1.02	0.00	(1.01–1.02)
Female 60–69	1.02	0.00	(1.01–1.03)	1.02	0.09	(1.00–1.04)	1.02	0.00	(1.01–1.03)
Female 70–79	1.01	0.00	(1.01–1.02)	1.02	0.01	(1.00–1.03)	1.01	0.00	(1.01–1.02)
Female 80+	1.01	0.00	(1.00–1.01)	1.00	0.70	(0.99–1.01)	1.01	0.00	(1.01–1.01)
Proportion of Population by race									
White	1			1			1		
Black	0.91	0.00	(0.88–0.95)	0.81	0.00	(0.73–0.89)	0.93	0.00	(0.90–0.97)
East/Southeast Asian	0.91	0.00	(0.90–0.92)	0.88	0.00	(0.86–0.91)	0.92	0.00	(0.90–0.93)
South Asian	0.88	0.00	(0.87–0.90)	0.90	0.00	(0.86–0.94)	0.88	0.00	(0.86–0.90)
Other groups	0.98	0.09	(0.95–1.00)	0.99	0.69	(0.92–1.05)	0.98	0.16	(0.95–1.01)

*CI* confidence interval, *IRR* incidence rate ratio

^a^Negative binomial regressions on the incidence of diagnosed cases of melanoma by dissemination area (DA) and census year. Table presents regression results for control variables not reported in Table 1

^b^Cases of in situ melanoma are not available for Ontario in the Canadian Cancer Registry dataset in the Statistics Canada Research Data Centre so Ontario DAs are excluded from these regressions

### Incidence by socioeconomic status

Table 1 shows that, for invasive melanoma, individuals in the lowest InQ had an incidence rate that was 82 % of the incidence rate of individuals in the highest InQ (IRR 0.82, *p* < 0.000, 95 % CI 0.78–0.85) after controlling for age, sex and race distribution and other factors. A similar, but stronger, association existed for melanoma in situ; again, a progressively lower incidence rate ratio was identified for progressively lower InQs, with the lowest InQ having an incidence rate that was 68 % of that for the highest InQ (IRR 0.68, *p* < 0.000, 95 % CI 0.62–0.73). The analysis was repeated with the Ontario data and showed no significant difference from the results excluding Ontario. In the first column of results from Table 1, determinants of the diagnosis of both invasive and in situ melanoma for all provinces and territories except Ontario (as in situ data was not available for Ontario) were estimated. Progressively lower InQs were associated with a progressively lower diagnosis of melanoma and the IRR of each InQ relative to the highest was significantly <1.

### Incidence by province of residence

There were wide disparities in the incidence rate of melanoma across the various provinces of Canada, as seen in Table 1. British Columbia was used as the reference province. Ontario was omitted to facilitate comparisons between in situ and invasive melanoma (only results for invasive melanoma in Ontario were available). Again, age, sex and race were controlled for multivariate regression analysis. For invasive melanoma, several provinces had IRRs that were significantly less than for British Columbia, with the most significant difference occurring in the province of Quebec, with an incidence rate only 44 % of that for the reference province (IRR 0.44, *p* < 0.000, 95 % CI 0.42–0.45). Other provinces with significantly lower IRRs included Newfoundland (IRR 0.66, *p* < 0.000, 95 % CI 0.61–0.71), Manitoba (IRR 0.74, *p* < 0.000, 95 % CI 0.70–0.78), Saskatchewan (IRR 0.75, *p* < 0.000, 95 % CI 0.70–0.80), Alberta (IRR 0.85, *p* < 0.000, 95 % CI 0.82–0.89) and the Territories (IRR 0.43, *p* < 0.000, 95 % CI 0.33–0.56). For melanoma in situ, different provincial patterns exist; several provinces had IRRs significantly greater than British Columbia, including Prince Edward Island, New Brunswick, Nova Scotia and Alberta, while Quebec, Saskatchewan and the Territories had IRRs markedly less than reference (e.g., Quebec [IRR 0.17, *p* < 0.000, 95 % CI 0.15–0.18]).

### Incidence by urban/rural residence

Table 1 demonstrates the results of our analysis of melanoma incidence by residence population density. For invasive melanoma (again, excluding Ontario, but controlling for age, sex and race), individuals living in rural areas had incidence rates significantly less than individuals in cities (IRR 0.82, *p* < 0.000, 95 % CI 0.79–0.85). Similar results were seen for individuals living in towns (IRR 0.82, *p* < 0.000, 95 % CI 0.79–0.85). Again, similar, but more dramatic, differences were seen for in situ melanoma; for rural residence, the incidence rate was 71 % of that for cities (IRR 0.71, *p* < 0.000, 95 % CI 0.67–0.75) and for towns, the incidence rate was 69 % of that for cities (IRR 0.69, *p* < 0.000, 95 % CI 0.65–0.74). Reintroducing Ontario into the analysis did not significantly change these results.

## Discussion

Our study confirmed that within Canada, like in other developed countries, the incidence of melanoma has risen dramatically in the 15 years spanned by these data [1–7]. Interestingly, our data showed that the rate of increase in melanoma incidence has accelerated between the 2001 and 2006 CYs. Multiple factors have been attributed to this overall rise in melanoma incidence, including depletion of the ozone layer (and its attendant protection from solar ultraviolet-B radiation) [1]. In addition, societal attitudes toward tanning have changed over the past several decades, with an increased association between tanned skin and physical attractiveness. The availability of tanning beds, and the exposure to them among young people, has also been associated with an increase in the incidence of melanoma [11, 12]. An increase in leisure time in developed societies is thought to have led to more vacations spent in southern climes as well as an increase in outdoor tanning [22]. The increased aging of our population also is likely a factor in higher melanoma rates [17, 18].

Other authors have pointed to changing criteria for the diagnosis of melanoma, which have increased the number of melanomas being diagnosed. Weyers et al. refers to this increase in melanoma incidence as a “pseudoepidemic,” and argues that melanomas are being detected now that would otherwise have regressed naturally [28]. An increased awareness about melanoma screening has led to cases being diagnosed at earlier stages; this was illustrated in our data by the fact that the increase in incidence for melanoma in situ was substantially larger than that of melanoma as a whole.

The second finding in our study was a strong association between higher SES and increased melanoma incidence. This association between high SES and higher incidence of melanoma is likely also multifactorial, as many of the explanations for the increased incidence of melanoma may impact individuals of different socioeconomic status differently [10, 19–21, 24]. Factors like access to tanning beds, vacation travel to warmer climates and the availability of leisure time, which may be spent sunbathing, are plausibly more prevalent in individuals from more advantaged socioeconomic circumstances. It is even plausible that attitudes about physical attractiveness and sun tanning are different between different socioeconomic strata. Many studies have also shown that awareness of, and access to, screening for cancer is disproportionately higher in individuals with higher SES [29]. Individuals of higher SES may be more likely to see a dermatologist and to investigate abnormal pigmented lesions. It should be noted that the effects of race on SES and melanoma incidence may represent an important confounder; we did attempt to eliminate the possibility of such confounding by including race in our logistic regression analysis.

The third finding in our study was a higher incidence of melanoma, even when controlling for SES and other factors, in urban residents of Canada. Our own previous study with thyroid cancer, another malignancy frequently detected during screening exams, found that urban residence (rather than in towns or rural areas) correlates with increased detection of cancer in Canada [26]. This may be due to access to a physician in general or more specifically to a dermatologist. Di Quinzio et al found that family physician visits correlated with earlier stage melanoma [30]. Certainly, dermatologists are typically concentrated in urban centers in Canada, and patients may be more likely to be referred to a specialist such as a dermatologist regarding suspicious pigmented lesions if they live in an urban area. Whether individuals who live in cities spend more time in the sun than town or rural residents is unclear at present.

Finally, our study found substantial discrepancies in melanoma incidence across different Canadian provinces. This is similar to what was previously found by Gaudette and Gao [31]. Further studies are needed to help elucidate the reasons for this large discrepancy, but access to screening for melanoma and access to specialists such as dermatologists may also differ from province to province, in the same way that they differ for urban and rural residences.

Our study had several important limitations. Not all data for the incidence of melanoma was available in some provinces, and in situ data was not available for Ontario. Second, while we did control for race using the techniques described, race was not included in the Canadian Cancer Registry data. Thirdly, we similarly do not possess data within the Canadian Cancer Registry on such important characteristics as tanning bed use, time spent in sun or awareness of/access to screening tests for melanoma on the individual level. Finally, our data is only up until 2006, as the long form census was not used in 2011.

## Conclusions

The incidence of melanoma rose significantly in our study from 1992 to 2006; this rise was most striking in melanoma in situ. Individuals with higher SES and patients in urban centers had significantly higher incidence rates of melanoma than individuals with lower SES or who resided in towns or rural areas. Finally, there were differences, in some cases quite dramatic, between the various provinces of Canada with respect to melanoma incidence.

## Abbreviations

CY: census year
DA: dissemination area
InQ: income quintile
NB-RDC: New Brunswick Research Data Centre
SES: socioeconomic status
